# SOCIAL CONNECTION IN OLDER ARKANSANS DURING COVID-19: CHANGES AND SATISFACTION

**DOI:** 10.1093/geroni/igac059.1962

**Published:** 2022-12-20

**Authors:** Robin McAtee, Jennifer Peszka, Anne Goldberg, Laura Spradley, Noura Musallam, Lauren Allen, Sophie Vogle, Luke Simpson

**Affiliations:** University of Arkansas for Medical Sciences, Little Rock, Arkansas, United States; Hendrix College, Conway, Arkansas, United States; Hendrix College, Conway, Arkansas, United States; University of Arkansas for Medical Sciences, Little Rock, Arkansas, United States; Hendrix College, Conway, Arkansas, United States; Hendrix College, Conway, Arkansas, United States; Hendrix College, Conway, Arkansas, United States; Hendrix College, Conway, Arkansas, United States

## Abstract

Social support is important to the health and well-being of older adults. Changes in social support (both frequency and satisfaction with) and barriers to social support during the pandemic remain unknown. During Spring 2021 an automated phone survey of adults 65 and older in Arkansas was completed. Calls were placed to 27,296 households where 8,745 individuals answered, and 867 (N=723 White, non-Hispanic) older Arkansans completed the 18 question self-report survey. Results indicated that: 41% had curtailed their in-person interactions significantly; 61% continued to engage in in-person social interactions outside of their home once a week or more; and social group activities decreased from 52% to 41%. Additionally, it was reported that 88% used technology for social interactions once a week or more and 60% reported technology interactions made them feel socially connected. Despite this, only 67% reported being satisfied with their social connection during the pandemic compared to 93% prior to the pandemic. While many participants continued in-person social interactions, social activity and satisfaction decreased during the pandemic. Social technology alternatives were used by many and for some, social connection was reported to be satisfactory. The value of what was learned from this survey has application outside pandemic times. Understanding and acknowledging that social isolation exists for older adults in normal times and improving technological access to social activities has great value. This knowledge can be used to substantiate the expansion and improvement of older adult friendly virtual platforms therefore contributing to reducing social isolation.

